# Exercise sensitizes PD-1/PD-L1 immunotherapy as a hypoxia modulator in the tumor microenvironment of melanoma

**DOI:** 10.3389/fimmu.2023.1265914

**Published:** 2023-10-09

**Authors:** Huiyu Yan, Aimin Jiang, Yinong Huang, Jun Zhang, Wenguang Yang, Wei Zhang, Tianya Liu

**Affiliations:** ^1^ National & Local Joint Engineering Research Center of Biodiagnosis and Biotherapy, The Second Affiliated Hospital of Xi’an Jiaotong University, Xi’an, China; ^2^ Center for Physical Education, Xi’an Jiaotong University, Xi’an, China; ^3^ Department of Medical Oncology, The First Affiliated Hospital of Xi’an Jiaotong University, Xi’an, China; ^4^ Shaanxi Institute of Pediatric Diseases, Xi’an Children’s Hospital, Xi’an, China; ^5^ Department of Talent Highland, The First Affiliated Hospital of Xi’an Jiaotong University, Xi’an, China; ^6^ Military Physical Education Teaching and Research Section of Air Force Medical Service Training Base, Air Force Medical University, Xi’an, China; ^7^ Institute for Stem Cell & Regenerative Medicine, The Second Affiliated Hospital of Xi’an Jiaotong University, Xi’an, China

**Keywords:** tumor microenvironment, exercise, hypoxia, PD-1/PD-L1 immunotherapy, immunotherapy sensitization

## Abstract

**Introduction:**

Hypoxia is associated with unfavorable prognoses in melanoma patients, and the limited response rates of patients to PD-1/PD-L1 blockade could be attributed to the immunosuppressive tumor microenvironment induced by hypoxia. Exercise offers numerous benefits in the anti-tumor process and has the potential to alleviate hypoxia; however, the precise mechanisms through which it exerts its anti-tumor effects remain unclear, and the presence of synergistic effects with PD-1/PD-L1 immunotherapy is yet to be definitively established.

**Methods:**

We established a B16F10 homograft malignant melanoma model and implemented two distinct exercise treatments (low/moderate-intensity swim) based on the mice’s exercise status. The specific function manner of exercise-induced anti-tumor effects was determined through RNA sequencing and analysis of changes in the tumor microenvironment. Furthermore, moderate-intensity swim that exhibited superior tumor suppression effects was combined with Anti-PD-1 treatment to evaluate its *in vivo* efficacy in mouse models.

**Results:**

Exercise intervention yielded a considerable effect in impeding tumor growth and promoting apoptosis. Immunohistochemistry and RNA sequencing revealed improvements in tumor hypoxia and down-regulation of hypoxia-related pathways. Cellular immunofluorescence and ELISA analyses demonstrated a notable increase of cytotoxic T cell amount and a decrease of regulatory T cells, indicating an improvement of tumor immune microenvironment. In comparison to Anti-PD-1 monotherapy, tumor suppressive efficacy of exercise combination therapy was found to be enhanced with improvements in both the hypoxic tumor microenvironment and T cell infiltration.

**Conclusion:**

Exercise has the potential to function as a hypoxia modulator improving the tumor immune microenvironment, resulting in the promotion of anti-tumor efficacy and the facilitation of biologically safe sensitization of PD-1/PD-L1 immunotherapy.

## 1 Introduction

Malignant melanoma, known for its elevated mortality rates and aggressive nature, presents a substantial burden that continues to escalate annually, thereby emerging as a pressing global public health issue (1, 2). Despite the encouraging outcomes of immune checkpoint blockade-based immunotherapies, such as PD-1/PD-L1 blockade, a significant proportion of individuals diagnosed with metastatic melanoma fail to respond to it. Moreover, those who do exhibit a response may encounter challenges associated with drug resistance and adverse reactions (3–5). Hypoxia, a prominent characteristic of the tumor microenvironment (TME), is a significant contributing factor to the inadequate treatment response observed in numerous solid tumors and is also counted as the culprit of immune evasion (6). Hypoxia arises as a consequence of aberrant metabolic processes within tumor cells, including heightened glycolysis and mitochondrial respiration (7). The resulting metabolites may participate in the mechanism of PD-L1 expression, thereby impeding T cell activation as well as bolstering the resistance of tumor cells against T cells (8, 9). In this manner, the presence of hypoxic TME hinders the functionality of anti-tumor immune cells and effectors, resulting in the establishment of an immunosuppressive microenvironment (10, 11). Consequently, the amelioration of hypoxia holds significant value in augmenting the response rates and durability of melanoma treatment efficacy. There has been a notable increase in the development of therapeutic interventions that specifically target the hypoxic microenvironment of tumors, which holds significant promise for achieving successful outcomes (12–14).

With the advancement of medical care and the escalating global population of individuals with tumors, there has been a heightened focus on the prognosis and adverse effects associated with cancer therapies. As an adjunctive treatment, exercise offers a non-invasive and non-toxic approach that allows for controlled intensity and effect, with a beneficial impact on mitigating the adverse effects of anti-tumor therapy, particularly in enhancing cardiorespiratory function (15–18). In addition to the fundamental impacts of exercise on enhancing overall well-being and reinstating bodily functionality, the advantages of exercise in combating tumors are evident. At the molecular level, exercise has been shown to increase the expression levels of anti-oncogenic and pro-apoptotic proteins, while disrupting the abnormal inhibition of apoptotic signaling (19, 20). At the cellular level, exercise has been observed to activate or regulate immune cells in TME, specifically including CD8^+^ T cells, CD4^+^ T cells and NK cells (21, 22). Reassuringly, tumor suppressive function of exercise has been observed in patients with various tumors and in preclinical mouse models (23–26). However, the challenge of developing exercise prescriptions for cancer patients is substantial due to the necessity of different anti-tumor therapeutic modalities, which may have adverse effects impacting exercise tolerance and previous therapeutic outcomes (15, 27). Hence, it is imperative to validate the *in vivo* synergistic effect of exercise and thoroughly analyze its underlying anti-tumor mechanisms.

Motivated by the role of exercise in tumor suppression and enhancement of cardiorespiratory function, the integration of exercise with alternative tumor treatment has garnered increasing attention. Researches have substantiated that the amalgamation of exercise intervention/rehabilitation with chemotherapy or radiotherapy yielded effective tumor control and physical condition improvement in patients (17, 18, 28, 29). Conversely, the combined approach of exercise and immunotherapy remains an area with numerous unresolved inquiries. Insufficient tumor antigen presentation and T cell exhaustion are significant contributors to immune evasion in melanoma; however, it is important to note that Anti-PD-1 monotherapy does not effectively modulate antigen presentation or reverse T cell status (30–32). In addition, the inappropriate combination of exercise with immune checkpoint inhibitors (ICIs) has the potential to increase the risk of immune-related adverse events (33). While preclinical mouse models of melanoma have yielded intriguing findings regarding the combination of exercise and ICIs, the underlying biological mechanisms remain poorly understood (34–36).

To explore the precise mechanisms by which exercise impacts tumor growth *in vivo*, we initially identified potential avenues for mechanistic investigations through clinical data analysis. Subsequently, we established a B16F10 homograft malignant melanoma model using C57BL/6 mice and implemented two distinct exercise trainings (low/moderate-intensity swim) based on the mice’s exercise status. Notably, exercise interventions exhibited tumor growth suppression and enhanced tumor cell apoptosis, among which the anti-tumor effect of the moderate-intensity swim was particularly pronounced. Results of immunohistochemistry and RNA sequencing revealed tumor hypoxia amelioration and the pathways associated with hypoxia were down-regulated in mice subjected to the exercise intervention. Consistently, cellular immunofluorescence and ELISA analyses indicated a notable augmentation in cytotoxic T cell population and a reduction in regulatory T cell count, thereby suggesting elevated TME immune conditions. The utilization of the selected moderate-intensity swim intervention in conjunction with Anti-PD-1 yielded notable results. Specifically, in comparison to Anti-PD-1 monotherapy, the tumor suppression efficacy of combined therapy was enhanced with a significant improvement in both hypoxia TME and T cell infiltration. Collectively, this work has shed light on the anti-tumor mechanism of exercise as a TME hypoxia modulator, and furnished a valuable point of reference for the implementation of exercise in combination with immunotherapy.

## 2 Results

### 2.1 Hypoxia has been linked to inferior overall survival and impaired immune function in melanoma

**Figure 1:**
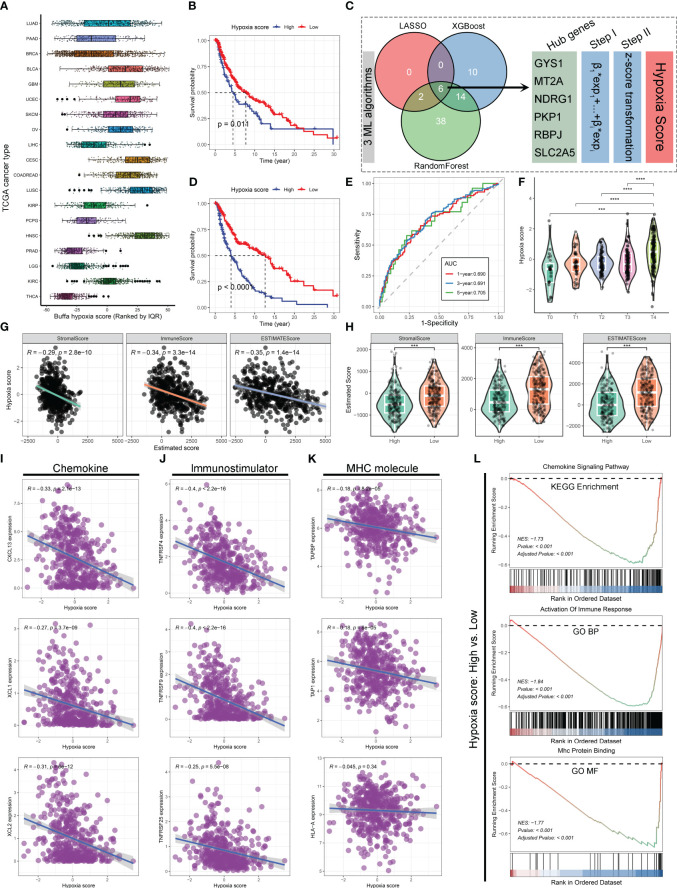
Hypoxia has been linked to inferior overall survival and impaired immune function in SKCM. **(A)** Boxplot illustrating the distribution of Buffa hypoxia scores among different cancer types in the TCGA database. The hypoxia scores were ranked using quantiles. **(B)** Kaplan-Meier survival curve demonstrating the overall survival (OS) of patients in the high and low hypoxia score groups based on Buffa hypoxia score. **(C)** Diagram illustrating the process of hypoxia score estimation based on 199 hypoxia-related genes using three machine learning algorithms in the TCGA-SKCM cohort. **(D)** Kaplan-Meier survival curve showing the OS of patients in the high and low hypoxia score groups based on the hypoxia scores derived from machine learning algorithms. **(E)** Time-dependent receiver operating characteristics (ROC) curves depicting the performance of machine learning algorithms derived hypoxia score in predicting 1-, 3-, and 5-year OS for patients with SKCM in the TCGA cohort. **(F)** Violin plot displaying the relationship between AJCC-T stage and hypoxia score in the TCGA-SKCM cohort. **(G, H)** Correlation scatter plots **(G)** and violin plots **(H)** demonstrating the associations between hypoxia scores and tumor stromal score, immune score, and estimated score. **(I–K)** Correlation scatter plots depicting the associations between hypoxia scores and the expression levels of chemokine **(I)**, immunostimulator **(J)**, and MHC molecules **(K)**. **(L)** GSEA revealing the alterations in the chemokine signaling pathway, immune response, and MHC protein binding between the high and low hypoxia score groups. ***, p<0.001; ****, p<0.0001.

Drawing inspiration from the hypoxia-immune microenvironment relationship, the initial investigation involved an evaluation of The Cancer Genome Atlas (TCGA) database’s pan-cancer data to determine the hypoxia status. (**Figure 1A**) It was revealed that the TCGA skin cutaneous melanoma (SKCM) tumor samples exhibited elevated Buffa hypoxia scores (37), suggesting melanoma could be classified among the cancers characterized by a heightened level of hypoxia. Further, 459 SKCM samples were categorized into two groups: ones with high hypoxia scores and the others with low hypoxia scores. Kaplan-Meier survival curves in the TCGA-SKCM cohort indicates that higher Buffa hypoxia scores are associated with a decreased overall survival (OS). (**Figure 1B**) In order to enhance the understanding of the associations between hypoxia and other relevant factors, three machine learning algorithms (LASSO regression, XGBoost, and RandomForest) were employed to ascertain pivotal hypoxia genes based on 199 hypoxia-related genes. Ultimately, six hub genes were identified by intersecting the common variables among the three machine learning algorithms. (**Figure 1C**) The reliability of the new hypoxia scoring approach based on six key genes was demonstrated by the pronounced difference in OS comparing the high hypoxia score group with low score group. (**Figures 1D, E**) Additionally, a strong correlation was identified between high hypoxia scores and tumor, node, metastasis (TNM) staging results of The American Joint Committee on Cancer (AJCC) in melanoma (**Figure 1F**), implying a potential link between heightened hypoxia levels and increased tumor invasiveness and metastatic dissemination (38, 39).

The potential association between hypoxia and the immune microenvironment status has been directly investigated in melanoma. Findings obtained from ESTIMATE Algorithm (40) application revealed a notable inverse relationship between hypoxia and Stroma score, Immune score, and Estimated score, indicating a compromised immune microenvironment infiltration. (**Figures 1G, H**) In order to provide additional evidence regarding the influence of hypoxia on the formation of the immunosuppressive tumor microenvironment, analyses were conducted between hypoxia and various non-cellular constituents within the tumor immune microenvironment. There was a notable correlation observed between higher hypoxia scores and lower expression levels of chemokine, immunostimulator, and major histocompatibility complex (MHC) molecules (**Figures 1I–K**), which play crucial roles in the anti-tumor immune response (41, 42). It was consistently supported by the findings of Gene Set Enrichment Analysis (GSEA), which indicated that the group with high hypoxia scores exhibited significant inhibitions of the chemokine signaling pathway, immune response activation, and MHC protein binding, as compared to the one with low hypoxia scores. (**Figure 1L**) According to these results, hypoxia amelioration presents a viable approach to enhance immune responses against melanoma. Considering the favorable impact of exercise on cardiorespiratory, it is worthwhile to investigate whether the improvement of hypoxia contributes to the mechanism of exercise-induced anti-tumor effects.

### 2.2 Exercise demonstrated evident anti-tumor effects and favorable biosafety in B16F10 homograft malignant melanoma model

**Figure 2:**
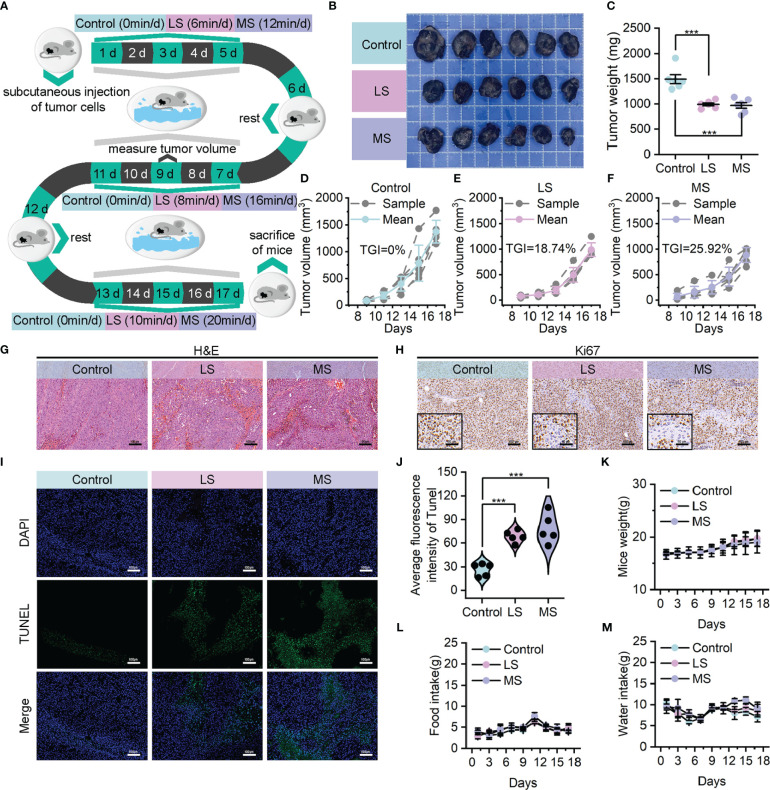
Exercise demonstrated evident anti-tumor effects in B16F10 homograft malignant melanoma model. **(A)** Model construction diagram for exercise treatment. **(B, C)** Tumor images **(B)** and corresponding tumor weights **(C)** following mentioned treatments. **(D–F)** Growth curves of tumor volumes in control **(D)**, LS **(E)** and MS **(F)** groups. **(G, H)** Upon completion of the experiment, using H&E **(G)** and Ki67 **(H)** to stain the resected tumors (scale bar: 100 μm, enlarged drawing: 50 μm). **(I, J)** TUNEL immunofluorescence images **(I)** and average fluorescence intensity **(J)** of tumor sections from mice following mentioned treatments. (scale bar: 100 μm, selected area: 5). **(K–M)** Weight **(K)** food intake **(L)** and water intake **(M)** of mice. The data were presented as mean ± s.d. Statistical significance of the differences between groups was determined using t test. ***, p<0.001.

In order to ascertain the credibility and dependability of exercise as an anti-tumor treatment, we established B16F10 homograft malignant melanoma model utilizing C57BL/6 mice. These mice were subjected to a 17-day intervention of weight-bearing swimming exercise with varying levels of intensity, while the weight of the load was determined as 5% of their own body weight based on their floating time. (**Supplementary Figure S1A**) Specifically, the mice were allocated in a random manner into three groups (n=6 per group): control, low-intensity swim (LS), and moderate-intensity swim (MS) groups, and then subjected to these exercise interventions with the same exercise cycle (1 day of rest for every 5 days of exercise) and different total exercise durations (0, 6/8/10, and 12/16/20 minutes per day). (**Figure 2A**) The variations in the overall level of physical activity among the distinct groups of mice were evidenced by the levels of lactate dehydrogenase (LDH) activity and lactate concentrations in blood. (**Supplementary Figures S1B, C**)

In contrast to the control group, the tumor photographs (**Figure 2B**) as well as tumor weights (**Figure 2C**) demonstrated reductions in tumor sizes among the LS and MS groups, suggesting that both exercise interventions were moderately successful in suppressing tumor growth. Additionally, the corresponding tumor volume curves provided further evidence to substantiate the certain degree of anti-tumor effectiveness, revealing a superior tumor growth inhibition value (TGI) in the MS group (25.92%) compared to the LS group (18.74%). (**Figures 2D–F**) As shown in hematoxylin & eosin (H&E) staining results (**Figure 2G**), the morphological integrity of tumor tissue was partially disturbed in both the LS and MS groups, and the severity of necrosis was notably higher in the MS group. In accordance with this, the results of Ki67 immunohistochemistry (**Figure 2H**), as well as Terminal deoxynucleotidyl transferase dUTP nick end labeling (TUNEL) staining (**Figures 2I, J**), revealed that the exercise intervention effectively suppressed tumor cell proliferation and facilitated apoptosis. Notably, there was hardly any significant difference in the body weights (**Figure 2K**) and food/water intake (**Figures 2L, M**) of the mice in LS/MS group compared with those in control group, demonstrating that the exercise treatments did not adversely affect their survival status. Simultaneously, neither moderate nor low intensities of exercise interventions resulted in substantial hepatic impairment or nephrotoxicity, while the impacts on blood, heart, lung and spleen were nearly inconsequential. (**Supplementary Figures S2**-**5**) These findings imply that exercise possesses biological safety as a therapeutic modality for cancer.

### 2.3 Exploring the underlying anti-tumor mechanism: exercise improved tumor immune infiltration through hypoxia alleviation

**Figure 3:**
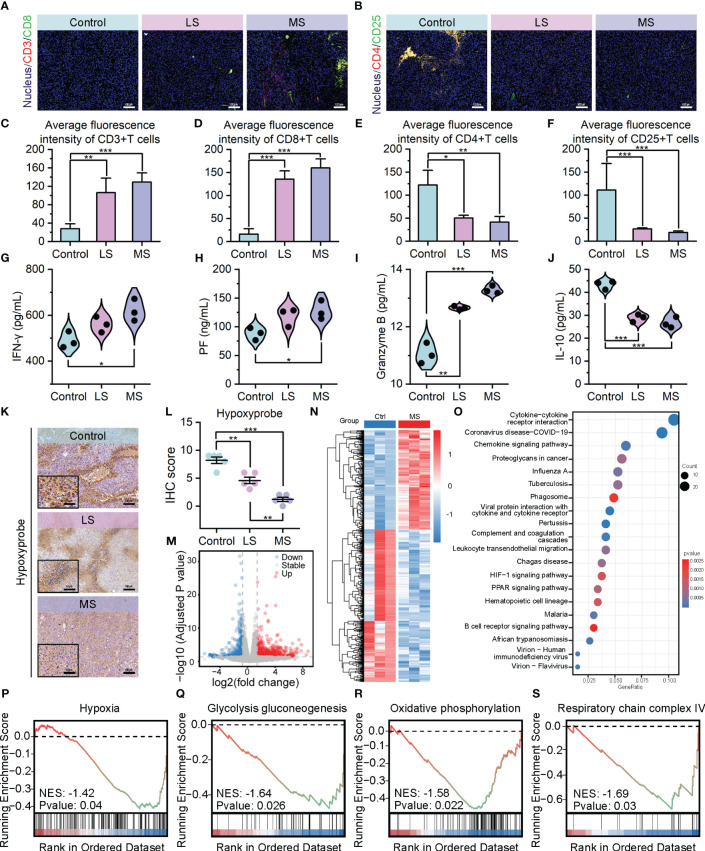
Exercise improved tumor immune infiltration through hypoxia alleviation. **(A–F)** Immunofluorescence images of CD3+/CD8+ cells **(A)** and CD4+/CD25+ cells **(B)**, and their average fluorescence intensity **(C–F)** of mice tumor sections following mentioned treatments (scale bar: 100 μm). **(G–J)** ELISA results of IFN-γ **(G)**, PF **(H)** Granzyme B, **(I)**, and IL-10 **(J)** levels in tumors. **(K, L)** Hypoxyprobe staining images **(K)** and IHC scores **(L)** of B16F10 tumor tissues in C57BL/6 mice with the specified treatments (scale bar: 100 μm, enlarged drawing: 50 μm; selected area: 5). **(M)** Volcano plot depicting the expression changes of 961 DEGs following MS intervention. **(N)** Hierarchal clustering heatmap illustrating the expression patterns of the identified 961 DEGs between the Ctrl and MS groups. Each group has three biological replication samples. The heatmap was clustered and scaled by the DEGs. **(O)** Dot plot highlighting the top 20 significantly enriched pathways identified through KEGG enrichment analysis. **(P–S)** GSEA revealing the significantly altered pathways or biological processes following MS intervention. The data were presented as mean ± s.d. Statistical significance of the differences between groups was determined using t test. *, p<0.05; **, p<0.01; ***, p<0.001.

To investigate the impact of exercise intervention on tumor development in the three groups, we conducted analyses of the tumor microenvironment. As depicted in **Figures 3A**, **C, D**, in contrast to the control group, a remarkable augmentation in the quantity of tumor-infiltrating cytotoxic T cells (CD3^+^/CD8^+^) has been observed in both LS and MS groups. Conversely, the population of regulatory T cells (CD4^+^/CD25^+^) has been reduced considerably after exercise administration. (**Figures 3B, E, F** and **Supplementary Figure S6**) Notably, these alterations in the T cell infiltration profile, which promote anti-tumor immune responses, were more pronounced in the MS group. In terms of anti-tumor immune cytokines, the exercise intervention groups, particularly MS group, exhibited a noteworthy elevation in interferon-γ (IFN-γ) levels in comparison to the control group, which represents an enhanced immune response (43). (**Figure 3G**) Elevated levels of perforin (PF) and granzyme B, the primary molecules responsible for T-cell-mediated cytotoxicity (44), were detected in both the LS and MS groups, aligning with the previously observed increase in apoptosis following exercise interventions. (**Figures 3H, I**) In contrast to the control group, the mice in LS and MS groups exhibited a significant decrease in interleukin-10 (IL-10) levels within their tumors (**Figure 3J**), potentially indicating an immune microenvironment enhancement and a partial alleviation of immunosuppression (45). Collectively, these data suggest that exercise positively affects the tumor immune microenvironment.

Considering the strong correlation between hypoxic TME and the immune response against tumors (**Figure 1**), we applied Hypoxyprobe-1 assay to compare the extent of hypoxia between the exercise and control groups. Consistent with our expectations, the exercise intervention led to an evident reduction in hypoxia levels within the tumor tissues of the mice. (**Figures 3K, L**) To delve deeper into the potential involvement of these findings in the anti-tumor mechanism of exercise, we utilized transcriptome sequencing (RNA sequencing) data to analyze the disparities in gene expression between the exercise and non-exercise groups. Volcano plot and hierarchical clustering heatmap presented the expression changes of 961 differentially expressed genes (DEGs) following MS intervention, among which 581 upregulated and 380 downregulated. (**Figures 3M, N**) Kyoto Encyclopedia of Genes and Genomes (KEGG) signaling pathway enrichment analysis showed that DEGs exhibited associations not only with signaling pathways pertaining to immune response and tumor metastasis, but also with metabolic pathways, particularly those related to hypoxia. (**Figure 3O**) The GSEA enrichment analysis exposed an inhibitory signal enrichment within hypoxia gene sets. (**Figure 3P**) Additionally, suppression features were enriched in the Glycolysis/Gluconeogenesis pathway (**Figure 3Q**), which is known to be positively regulated by hypoxia (46). Furthermore, there was also a prominent enrichment of suppressive signatures exhibited in gene sets related to oxidative phosphorylation and respiratory chain complex IV (**Figures 3R, S**), both of which are directly associated with oxygen utilization and its cellular level (47, 48). These results from RNA sequencing further confirmed the proactive role of exercise in hypoxia mitigation at transcriptional level. Collectively, our findings provide evidence that exercise enhances tumor immune infiltration and intratumoral immune responses through the modulation of the hypoxic TME, thereby exerting its anti-tumor efficacy.

### 2.4 Exercise achieves *in vivo* Anti-PD-1 immunotherapy sensitization by improving the immune microenvironment through hypoxia modulation

**Figure 4:**
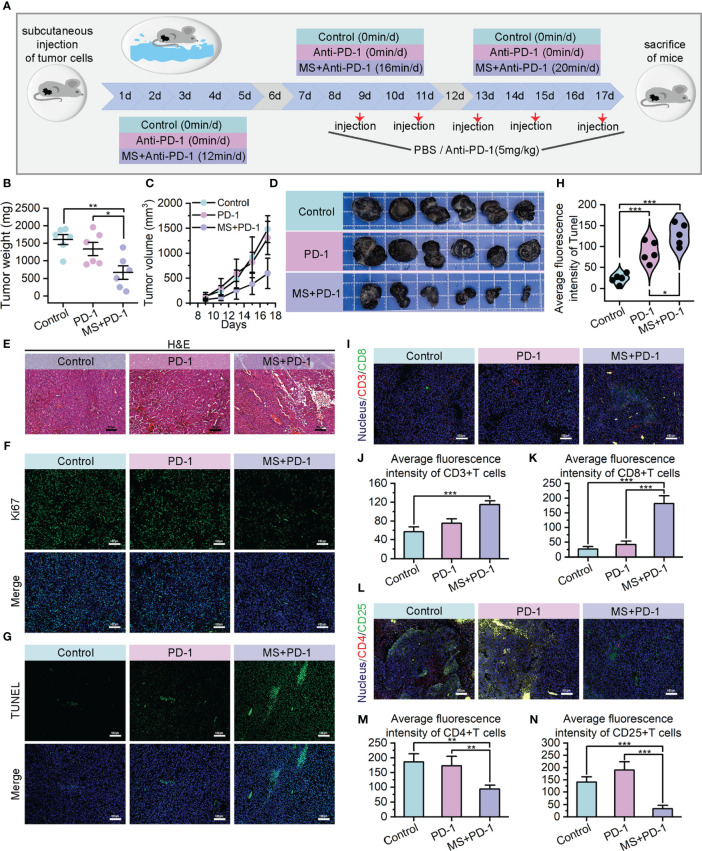
Exercise achieved *in vivo* Anti-PD-1 immunotherapy sensitization. **(A)** Schematic diagram of moderate intensity swim treatment and Anti-PD-1 combination therapy model construction. **(B–D)** Tumor weights **(B)**, growth curves of tumor volumes **(C)** and tumor images **(D)** of different treating groups. **(E)** H&E staining images of resected tumors (scale bar: 100 μm, enlarged drawing: 50 μm). **(F)** Immunofluorescence images of Ki67 in tumor sections from mice after different specified treatments (scale bar: 100 μm). **(G, H)** TUNEL immunofluorescence images **(G)** and average fluorescence intensity **(H)** of tumor sections. (scale bar: 100 μm, selected area: 5). **(I–K)** Immunofluorescence images of CD3+/CD8+ cells **(I)** and their average fluorescence intensity **(J, K)** in tumor sections of mice treated by different therapies (scale bar: 100 μm). **(L–N)** Immunofluorescence images of CD4+/CD25+ cells **(L)** and their average fluorescence intensity **(M, N)** in tumor sections of mice treated by different therapies (scale bar: 100 μm). The data were presented as mean ± s.d. Statistical significance of the differences between groups was determined using t test. *, p<0.05; **, p<0.01; ***, p<0.001.

PD-1/PD-L1 blockade is currently regarded as a foremost therapeutic approach for melanoma; nevertheless, it encounters challenges such as inadequate immune response and the emergence of drug resistance due to the intricate and suppressive characteristics of TME (49–51). The encouraging outcomes of exercise interventions on tumor immune responses prompt us to investigate the potential synergistic and sensitizing effects of exercise on PD-1/PD-L1 blockade by means of TME modifying. The B16F10 homograft malignant melanoma models were subjected to random assignment into three distinct groups: the PBS-treated group (Control), Anti-PD-1 monotherapy group (PD-1), and Exercise-Anti-PD-1 combination therapy group (MS+PD-1). The moderate-intensity swim treatment with superior anti-tumor performance was chosen for the exercise intervention, and the duration and frequency were consistent with the aforementioned details. Anti-PD-1 administration was performed via intraperitoneal injection with a dosage of 5 mg/kg every 2 days. (**Figure 4A**) The combination of exercise and Anti-PD-1 treatment exhibited remarkable *in vivo* therapeutic effects against tumors. In contrast to the control and Anti-PD-1 monotreatment cohorts, mice receiving combined treatment exhibited evident reduction in tumor weight and volume, indicating superior suppression of tumor growth. (**Figures 4B–D**) The efficacy of the combination therapy has also been supported by H&E staining from a pathological perspective. (**Figure 4E**) At the cellular level, the results of Ki67 immunofluorescence also indicated that tumor cell proliferation was suppressed with the intervention of exercise combined with Anti-PD-1. (**Figure 4F**, **Supplementary Figure S10A**) Meanwhile, the administration of combination therapy resulted in a remarkably higher quantity of apoptotic tumor cells compared to the administration of single Anti-PD-1, providing further evidence of the enhanced anti-tumor efficacy achieved through the exercise intervention in conjunction with immunotherapy. (**Figures 4G, H**, **Supplementary Figure S10B**).

**Figure 5:**
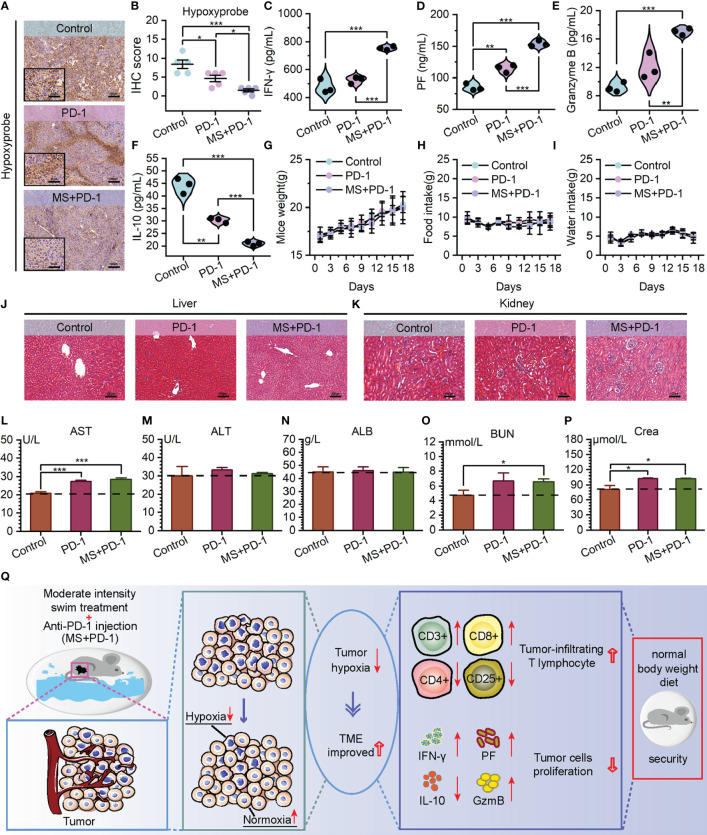
Exercise-Anti-PD-1 combined treatment alleviated TME hypoxia and improved the immune microenvironment with favorable biosafety. **(A, B)** Hypoxyprobe staining images **(A)** and IHC scores **(B)** of B16F10 tumor tissues in C57BL/6 mice with the specified treatments (scale bar: 100 μm, enlarged drawing: 50 μm; selected area: 5). **(C–F)** The detection of IFN-γ **(C)**, Perforin **(D)** Granzyme B **(E)**, and IL-10 **(F)** levels by ELISA in tumor tissues. **(G–I)** Weight **(G)** food intake **(H)** and water intake **(I)** of mice. **(J, K)** At the end of the experiment, using H&E to stain the liver **(J)** and kidney **(K)** from mice (scale bar: 100 μm). **(L–N)** Hepatotoxicity measured by aspartate aminotransferase (AST) **(L)**, alanine aminotransferase (ALT) **(M)**, and albumin (ALB) **(N)**. **(O, P)** Nephrotoxicity measured by blood urea nitrogen (BUN) **(O)** and creatinine (Crea) **(P)**. **(Q)** The schematic diagram of tumor reaction process after MS and Anti-PD-1 combination treatment. The data were presented as mean ± s.d. Statistical significance of the differences between groups was determined using t test. *, p<0.05; **, p<0.01; ***, p<0.001.

The noteworthy tumor growth suppression achieved by the exercise intervention combined with Anti-PD-1 emphasizes our need to investigate alterations within TME. Encouragingly, cytotoxic T cell infiltration exhibited a substantial increase in the tumor of the combined treating group, as opposed to Anti-PD-1 monotherapy and control groups. (**Figures 4I–K**) Meanwhile, a statistically notable decrease was found in the quantity of regulatory T cells that infiltrated the tumors of combination treatment group. (**Figures 4L–N**, **Supplementary Figure S7**) These observations potentially contribute to the immunosuppressive TME alteration. Prompted by this, we examined the presence of hypoxia in tumors across three cohorts. The data revealed that tumors derived from mice subjected to a combination of exercise and Anti-PD-1 treatment exhibited reduced hypoxia levels, reinforcing the association between hypoxia mitigation and immune microenvironment remodeling. (**Figures 5A, B**) Benefiting from the diminished degree of TME hypoxia, the levels of IFN-γ, perforin, and granzyme B within tumor tissues of mice subjected to combined treatment presented a noteworthy increase, and the levels of IL-10 experienced down-regulation when compared to control and mono-immunotherapy groups. (**Figures 5C–F**).

It is worth noting that hardly any alarming deleterious consequence has been observed in the tumor-bearing mice subjected to Exercise-Anti-PD-1 combined treatment. Throughout the treatment period and upon its culmination, the mice receiving combined therapy did not manifest abnormal weight reduction and maintained relatively consistent consumption of food and water in comparison to the mice of two remaining groups. (**Figures 5G–I**) Based on the evaluations of hepatotoxicity and nephrotoxicity, no further detrimental impacts on the standard hepatic and renal functioning were observed. (**Figures 5J–P**) Furthermore, the integration of exercise with immunotherapy did not cause evident adverse effects on the blood, heart, lung, and spleen. (**Supplementary Figures S8**, **9**) Taken together, these data presented in our study offer partial validation for the guideline’s assertion that there is a low incidence of exercise-related adverse events during treatment (52). Consequently, our results demonstrate that exercise, with a favorable biosafety profile, exerts synergistic anti-tumor effects with PD-1 blockade in a manner that improves TME hypoxia and augments the immune response. (**Figure 5Q**)

## 3 Discussion

It is widely acknowledged within the field of oncology that exercise holds positive implications for tumor treatment (53, 54). Presently, exercise has demonstrated numerous advantages in the management of various solid tumors, particularly those with a high prevalence, such as breast, lung, and colorectal cancers (52). However, variations in tumorigenesis and progression mechanisms, viable treatment options, and prognostic outcomes exist across distinct tumor types, necessitating comprehensive investigations into specific tumor types to yield more realistic data and treatment recommendations. For specific cancer types, the number of studies directly investigating the effects of two or more exercise intensities on tumor progression remains limited, with insufficient development of biological mechanisms. In this study, we implemented two exercise interventions, low-intensity and moderate-intensity swimming, tailored to the physical condition of mice, and examine the *in vivo* alterations induced by exercise on a B16F10 homograft malignant melanoma model. The findings obtained from animal and cellular analyses demonstrated both intensities of exercise interventions produced substantial inhibition of tumor growth and tumor cell proliferation, with moderate-intensity exercise intervention exhibited superior anti-tumor efficacy. The tumor tissues of mice that underwent exercise intervention presented a remarkable increase in CD3^+^/CD8^+^ T lymphocytes and a considerable decrease in CD4^+^/CD25^+^ T lymphocytes. Additionally, there was a notable increase in the levels of cytokines related to positive anti-tumor response, indicating an improvement of TME immune conditions and an enhancement of the anti-tumor immune response. Through immunohistochemistry and RNA sequencing, it is revealed that exercise intervention leads to a down-regulation of gene expression in hypoxia-related signaling pathways and subsequently alleviates tumor hypoxia, contributing to establishment of the more favorable physiological conditions for tumor immune microenvironment improvement. Consequently, we suggest that exercise acts as a hypoxia modulator in the anti-tumor process, inducing alterations in the immune microenvironment and ultimately resulting in a cascade of effects that include tumor cell elimination and inhibition of tumor growth.

Immunotherapy, specifically immune checkpoint inhibitors (ICIs) such as PD-1 antibodies, is the primary therapeutic approach for melanoma (4). Nevertheless, a significant proportion of melanoma patients, exceeding 60%, do not exhibit a satisfactory response to PD-1/PD-L1 blockade, and augmentation therapy with PD-1 antibodies in combination with CTLA-4 antibodies only enhances the response rate by a modest 20% (55–57). Patients who do not respond to these treatments, with low tumor immunogenicity and experience T cell exhaustion within the tumor microenvironment, encounter the challenge of limited therapeutic options. Additionally, patients who initially respond to ICIs also confront the issue of developing acquired resistance (58). Despite the existence of a diverse range of strategies aimed at augmenting the therapeutic efficacy of ICIs (59–63), the majority of these approaches are still in the preclinical phase. Motivated by the advancements in tumor hypoxic microenvironment enhancement and immune remodeling through single exercise intervention, we employed B16F10 cells characterized by high tumorigenicity and low immunogenicity (64, 65) to establish a B16F10 homograft malignant melanoma model. Subsequently, we integrated moderate-intensity exercise intervention, known for its superior anti-tumor efficacy in our previous test, with Anti-PD-1 administration to create a synergistic treatment protocol. The results indicated that the combination-treated mice had significantly lower tumor volume and weight, as well as a higher count of apoptotic tumor cells in comparison to the mice receiving mock treatment or Anti-PD-1 monotherapy. In the context of limited efficacy of single Anti-PD-1, it is noteworthy that the combination treating group exhibited reduced tumor hypoxia and displayed more pronounced positive outcomes, including heightened infiltration of cytotoxic T lymphocytes, diminished presence of regulatory T lymphocytes, and elevated levels of tumor-suppressing cytokines. Collectively, exercise sensitizes Anti-PD-1 treatment and synergistically leads to an anti-melanoma therapeutic effect, as demonstrated by the results at the animal and cellular levels. Given the controversial opinions of exercise-ICI combination therapy tested on preclinical models of melanoma, our findings might provide some evidence for the feasibility and efficacy of exercise interventions in combination with ICIs.

The benefits of exercise for tumor survivors are unquestionable, while there are two supplementary factors to consider when integrating moderate exercise into the comprehensive regimen of melanoma treatment. Primarily, due to the intricacy of diagnosing and treating tumor patients, it is crucial to guarantee the safety of exercise training in terms of physical functionality. Secondly, the emergence of novel therapies, such as immunotherapy, carries the potential for physiological adverse effects, thereby necessitating further investigation into the correlation between exercise interventions and these detrimental effects. During the concurrent administration of exercise and PD-1 blockade in this study, the mice body weights and food/water intaking status of the tumor-bearing mice remained relatively stable, and there were no abnormalities observed in the hepatotoxicity, nephrotoxicity, hematotoxicity tests and pathological sections of tissues. These results provide evidence for the biosafety of exercise intervention during tumor treatment. Furthermore, in light of advancements in oncology treatments and care protocols, it is imperative to emphasize the significance of cancer patients preserving their functional capacities and enhancing their overall well-being. Exercise, as a therapeutic modality aimed at augmenting physical fitness and ameliorating living conditions, plays a constructive role in alleviating symptoms such as tumor-related fatigue, thus warranting further researches. Based on our data indicating the positive impact of exercise on tumor hypoxia, future investigations could explore the feasibility of integrating exercise with other therapeutic modalities for tumors or extend the duration of monitoring in tumor models (16). In summary, this work presents evidence supporting the anti-tumor function of exercise, offers a detailed explanation of how exercise improves the tumor microenvironment and enhances the immune response against tumors by alleviating hypoxia and demonstrates the effectiveness and safety of exercise-enhanced PD-1/PD-L1 blockade, which shedding light on the positive role of exercise in tumor therapy and presenting a potential avenue for enhancing melanoma immunotherapy.

## Data availability statement

The original contributions presented in the study are included in the article/**Supplementary Material**. Further inquiries can be directed to the corresponding authors.

## Ethics statement

The animal study was approved by the Medical Ethics Committee of Xi’an Jiaotong University. The study was conducted in accordance with the local legislation and institutional requirements.

## Author contributions

TL: Conceptualization, Data curation, Funding acquisition, Project administration, Supervision, Writing – original draft, Writing – review & editing. HY: Data curation, Methodology, Project administration, Visualization, Writing – original draft. AJ: Data curation, Investigation, Methodology, Software, Validation, Visualization, Writing – review & editing. YH: Resources, Supervision, Writing – review & editing. JZ: Supervision, Writing – review & editing. WY: Data curation, Investigation, Methodology, Writing – review & editing. WZ: Supervision, Validation, Writing – review & editing.
