# Supplementary material for: Exercise sensitizes PD-1/PD-L1 immunotherapy as a hypoxia modulator in the tumor microenvironment of melanoma
Source: Front Immunol. 2023 Oct 9;14:1265914. doi: 10.3389/fimmu.2023.1265914 (PMC10590877; doi:10.3389/fimmu.2023.1265914)
Supplement: Supplementary file 1 [file DataSheet_1.docx]

Supplementary Material

# Materials and methods

## Cell culture and model of B16F10 melanoma

Mouse melanoma B16F10 cells (Chinese Academy of Science Cell Bank, China) were cultured in DMEM medium (Thermo Fisher Scientific, China) which was supplemented with 10 percent of fetal bovine serum (FBS). The culture environment was filled with 5 percent of CO_2_ and the temperature was maintained at 37℃. With the action of 0.05 percent of trypsin-EDTA (Thermo Fisher Scientific, China), sufficient B16F10 cells were collected and subcutaneously transplanted into unilateral buttocks of 4-5-week-old female C57BL/6 mice (5×10^5^ cells/100 μl PBS).

## Swimming intervention and testing

In this work, we chose swimming with load as the exercise treatment, which enables flexible controlling for the intensity of exercise. In accordance with the guidelines outlined in t*he Design Of Animal Exercise Protocols* (1), the swim training was conducted within a transparent bucket measuring 30 and 25 cm in height and diameter respectively. The bucket contained water with a depth of 20 cm and a temperature maintained at 31±1℃. Preliminary swimming experiments with different loads were performed. As shown in **Supplementary Figure S1A**, 5 percent of body weight is a proper load for the mice to bear, and thus was taken in all later experiments. In addition, the lactic acid levels in bloods were measured immediately after swim for the mice experiencing different time of swim, see in **Supplementary Figure S1B**. It indicated a positive correlation between swim time and lactic acid levels, suggesting that the duration of swimming could serve as an indicator of exercise intensity.

## Establishment of B16F10 homograft malignant melanoma model receiving swim treatment

Upon inoculation with B16F10 cells to mice, three training groups were created randomly: the control group (Control, n = 6), the low-intensity swim treatment group (LS, n = 6), and the moderate-intensity swim treatment group (MS, n = 6). The mice of LS and MS groups would take the swim treatment in a progressive training style for three phases. In the first phase, the mice of LS group trained on 6 min/day for five days and then rested for one day, while those of MS group trained on 12 min/day for five days and then rested for one day. In the second and third phases, the daily swim training duration for LS group was adjusted to 8 min/day and 10 min/day, respectively, and that for MS group was 16 min/day and 20 min/day, respectively. Correspondingly, the mice of control group were put into the same swimming environment where the water level did not affect the mice’s normal activity and respiration. In addition, throughout the entire experiment, the feed and water given to the mice were weighed and recorded at consistent intervals of two days. The final intake of both feed and water was calculated as the average intake of all the mice. The equation utilized for the calculation of tumor volume was as follows: tumor volume (V) = width^2^ × length × 1/2. The measurement of tumor volume was documented once the average tumor volume reached approximately 50 mm^3^. After a single swim training, the lactate dehydrogenase (LDH) activity in the blood of mice was immediately tested. As shown in **Supplementary Figure S1C**, the LDH activity of mice with swim treatment is clearly increased. In addition, the mice of MS group have significantly higher LDH activity than those of LS group. These results indicate that our progressive training modes for LS and MS groups can lead to distinguishable exercise intensities.

## Measurements of Lactic Acid activity

Blood samples of mice were first dealt with by using the Lactic Acid assay kit (Nanjing Jiancheng Bioengineering Institute, China) specifications. Then, the absorbance was measured at 530nm with a 1cm light diameter by the Enzyme-labeled instrument (Thermo Fisher Scientific, China).

## Measurements of LDH activity

The whole blood samples were taken from mice and placed in the anticoagulant tubes. Firstly, the whole blood with anticoagulant was centrifuged at 200 × g for 15 minutes to prepare serums. Then, the serums were handled in accordance with the LDH assay kit specifications (Solarbio Technology Co., Ltd, China), and the absorbance of each sample at 450 nm by using an Enzyme-labeled instrument.

## Establishment of B16F10 homograft malignant melanoma model receiving MS and Anti-PD-1 combined treatment

After being inoculated with B16F10 cells, mice were stochastically divided into three groups: the control group with injection of PBS (Control, n = 6), the Anti-PD-1 monotherapy group with Anti-PD-1 injection (PD-1, 5 mg/kg, n = 6), and the combination of moderate intensity swim treatment with Anti-PD-1 injection group (MS+PD-1, 5 mg/kg, n = 6). Both the Control and PD-1 groups were kept in shallow water environment. The training style of the MS+PD-1 group was the same as that of the MS group in *1.3*. The method used to record the food and water intake of the mice was also the same as in *1.3*. When the average tumor volume reached ~50 mm^3^, the PBS/Anti-PD-1 drugs were injected intraperitoneally every two days. The determination of tumor volume (V) was conducted through the utilization of the following equation: V= width^2^ × length × 1/2.

## Data acquisition

Clinical data and mRNA expression matrix of patients with skin cutaneous melanoma (SKCM) were obtained from the Cancer Genome Atlas (TCGA) database through the GDC data portal (https://portal.gdc.cancer.gov/) for subsequent analysis. The pan-cancer Buffa hypoxia scores, which assess the hypoxia status, were obtained from previously published studies (2, 3) to investigate the hypoxia status of TCGA-SKCM patients.

## The relationship between Buffa hypoxia score and OS among SKCM samples

Firstly, the Buffa hypoxia scores were ranked based on the interquartile range (IQR) among 19 different malignancy types in the TCGA database. Boxplots were utilized to visualize the ranking of Buffa hypoxia scores across these cancer types. Next, SKCM patients were categorized into groups with high or low hypoxia scores respectively using a predetermined cut-off value determined by the "surv_cutpoint" function from the R software's "survminer" package. Subsequently, Kaplan-Meier survival curves were conducted to assess the overall survival (OS) differences between two groups. The log-rank test was employed to evaluate survival differences’ statistical significance.

## Machine learning-assisted hypoxia score estimation on the basis of genes related to hypoxia

From the Molecular Signatures Database (MSigDB, https://www.gsea-msigdb.org/gsea/msigdb), the hallmark gene sets were requisitioned for 200 candidate genes related to hypoxia. To identify key hypoxia-related targets and calculate the hypoxia score in the TCGA-SKCM cohort, three machine learning algorithms were employed: Least Absolute Shrinkage and Selection Operator (LASSO) regression, XGBoost, as well as RandomForest. In LASSO regression, the hub hypoxia-related genes with non-zero coefficients were identified based on "lambda.1se". A list of the top 30 hypoxia-related genes was generated using the XGBoost function "xgb.importance". The key hypoxia-related genes were identified in RandomForest using the "var.select" function. Finally, among the three machine learning algorithms, the most important genes related to hypoxia were identified by intersecting common variables. After identifying the hub hypoxia-related genes, the hypoxia score was calculated. This involved performing multivariate Cox regression to obtain the coefficients of these hub genes. The hypoxia score for each patient was then estimated on the basis of the hub genes’ mRNA expression values and their corresponding coefficients. These hypoxia scores were further transformed using z-score normalization for comparability. Using the transformed hypoxia score, SKCM patients in the same cohort were divided into ‘high hypoxia score’ group if the score was greater than zero, and into the ‘low hypoxia score’ group otherwise.

## The prognostic significance of machine learning-assisted hypoxia score and its association with the tumor immune microenvironment

Kaplan-Meier survival curves were conducted to assess differences between OS of high machine learning-aided hypoxia score group and the one of low score group, with the log-rank test employed for statistical significance determination. Additionally, the performance of the machine learning-aided hypoxia score in predicting SKCM patients’ 1-, 3-, and 5-year OS was evaluated by time-dependent receiver operating characteristic (ROC) curves. Furthermore, violin plots were utilized to examine the association between the hypoxia score and the American Joint Committee on Cancer (AJCC)-T stage, with statistical differences estimated using the t-test.

The ESTIMATE algorithm, a well-established tool for inferring stromal and immune cells’ fraction in tumor samples (4), was employed to estimate the stromal score, immune score, and estimate score of SKCM patients based on the mRNA expression matrix. Correlation scatter plots and violin plots were generated to explore the relationships of the hypoxia scores with these scores. Subsequently, we investigated the associations between hypoxia scores estimated above and the chemokine signaling pathway (*CXCL13*, *XCL1*, and *XCL2*), immunostimulator molecules (*TNFRSF4*, *TNFRSF9*, and *TNFRSF25*), and major histocompatibility complex (MHC) molecules (*TAPBP*, *TAP1*, and *HLA-A*). Moreover, we performed Gene Set Enrichment Analysis (GSEA) to identify significantly altered signaling pathways or processes in comparation of high with low hypoxia score groups. Reference gene sets ("c5.go.v2023.1.Hs.symbols.gmt" , "c2.cp.kegg.v2023.1.Hs.symbols.gmt" ) were utilized, with a significance threshold set at P value < 0.05 and adjusted P value < 0.25. R software was employed for GSEA analysis conduction, specifically the "clusterProfiler" and "enrichplot" packages.

## RNA-seq analysis of murine tumor tissue

Differential analysis was conducted using R software "DESeq2" package, with raw count data used as the input file. The differentially expressed genes (DEGs) following MS intervention were ascertained according to the following criteria: |log2Foldchange (FC)| > 1 and adjusted P value < 0.05. These DEGs were classified as up-regulated genes if their log2FC > 1 and adjusted P value < 0.05 while down-regulated genes if their log2FC < -1 and adjusted P value < 0.05. The volcano plot and scaled hierarchical clustering heatmap were used to visualize DEG distribution and expression patterns between the Ctrl and MS groups, respectively.

To identify those significantly enriched pathways or processes impacted by MS intervention, we performed an analysis of pathway enrichment using the Kyoto Encyclopedia of Genes and Genomes (KEGG) database. The top 20 enriched pathways were visualized using a dot plot. Additionally, GSEA was utilized to explore the significantly altered pathways or processes between the Ctrl and MS groups. Mouse gene sets from the MSigDB database were employed as reference gene sets. The thresholds for the P value and adjusted P value in GSEA analysis were the same as those used in the aforementioned analysis.

## H&E and immunohistochemical (IHC) staining

At the end of the treatment, the tumor as well as major organs (liver, kidney, lung, spleen, heart) of mice were removed and then fixed in formalin. Hematoxylin & eosin (H&E) stain were used to deal with paraffin-embedded (FFPE) sections (4.0 μm each section). (5) For immunohistochemistry, the sections were incubated with Ki67 (CST, China) antibody overnight (4°C) and LSAB complex for 15 minutes. Finally, the slides were stained and visualized for analysis after 15 min of incubation with the LSAB complex. The representative images were obtained with a Nikon Eclipse Ni-U microscope (Nikon, Japan) through the NIS Elements imaging software. (6) In addition, five random regions were selected for each sample to measure the following-mentioned IHC score.

The intensity of immune staining (I) was evaluated by using the numerical score (0-3): 0-unstained, 1-weak staining, 2-moderate staining, and 3-strong staining. The immunostaining area (A) was assessed using numerical score (1-4) according to the proportion of positive area: 1-0% to 10%, 2-10% to 50%, 3-50% to 90%, and 4-90% to 100%. Accordingly, the immunohistochemical (IHC) score was calculated as the staining intensity score (I) multiplied by the staining area score (A), i.e. IHC score = I × A. (7)

## Immunofluorescence staining

Sections of tumors were stained with primary antibodies and the corresponding secondary antibodies coupled to Alexa Fluor-488 or Alexa Fluor-568. (5) Then, all sections were stained by vectashield mount medium containing DAPI. The main antibodies are as follows: TUNEL (Bioss, China), Anti-CD3 (Bioss, China) and Anti-CD8 (Bioss, China); Anti-CD4 (Santa Cruz, USA); Anti-CD25 (Abcam, USA). Images were obtained by Pannoramic MIDI Digital Slide Scanners (3DHISTECN, Hungary) and the quantitative histomorphometric analysis was conducted by Image-Pro Plus software on five random regions selected for each sample.

## Enzyme-linked immunosorbent assay

Enzyme-linked immunosorbent assay (ELISA) for tumor tissue homogenate samples were carried out by using paired antibody sets (Solarbio technology Co., Ltd, China). Interferon γ (IFN-γ), perforin (PF), granzyme-B, and interleukin-10 (IL-10) were measured according to the instruction of the manufacturer (Solarbio technology Co., Ltd, China).

## Hypoxia detection of tumor tissue

Approximately 100 μl of Hypoxyprobe-1 solution (60 mg per kg body weight) was administered via intraperitoneal injection to each mouse's abdominal cavity one hour prior to their sacrifice. After sacrificing the mice, the tumor tissues were removed and dealt with by routine fixation, embedding, and sectioning. The hypoxia in the tumor tissue was detected by the staining analysis, and the representative images were conducted by a Nikon Eclipse Ni-U microscope (Nikon, Japan) with the NIS Elements imaging software.

## Blood test analysis

Upon completion of the experimental intervention, fresh whole blood was obtained from mice and prepared into plasma and serum for blood routine, liver function and renal function test. The main indices included white blood cell (WBC), red blood cell (RBC), thrombocyte, aspartate aminotransferase (AST), albumin (ALB), alanine aminotransferase (ALT), creatinine (Crea) and blood urea nitrogen (BUN). Moreover, all blood tests were performed in accordance with standard clinical trial procedures of the Clinical Laboratory Department of Affiliated Hospital of Xi'an Jiaotong University.

## Ethics approval and consent to participate

The mice were subjected to a standardized feeding regimen and housed in a specifically controlled environment that was free from pathogens, with a regular light-dark cycle. All mouse experiments conducted in this study received approval from the Medical Ethics Committee of Xi'an Jiaotong University for Animal Ethics (protocol code 2021-1727) and were conducted in compliance with relevant regulations.

## Statistical analysis

The statistical analysis employed Two-sided Student's t-tests to compare two groups. A significance level of p < 0.05 was deemed statistically significant. Data were presented as mean ± s.d.

# Supplementary Figures


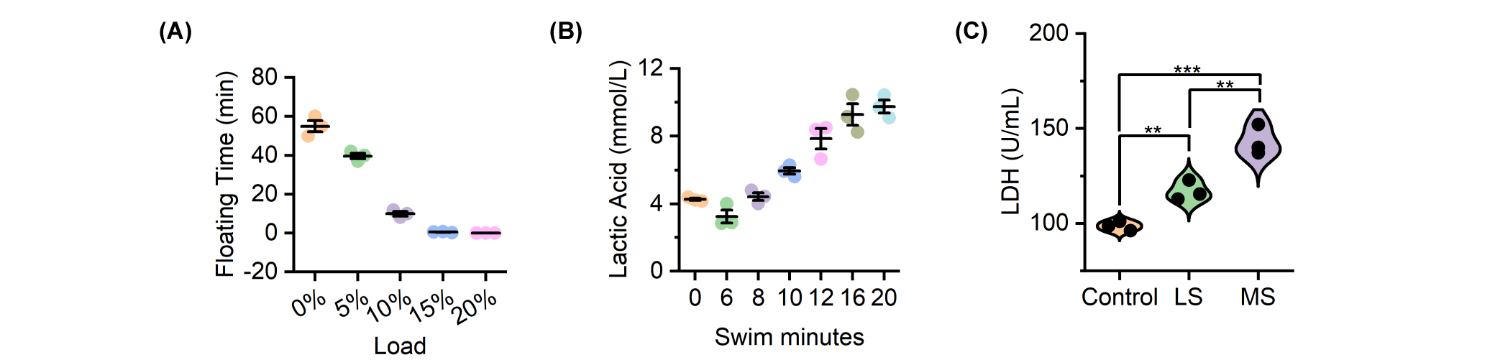


**Supplementary Figure 1.** (A) The floating times of tumor mice loaded with different weights of lead blocks (0%, 5%, 10%, 15%, and 20% of the body weight of the mouse). (B) Lactic Acid activity in the blood of mice swimming for different lengths of time. (C) Lactate dehydrogenase (LDH) activity in the blood of mice after swim treatment. The data were presented as mean±s.d. Difference between groups was examined for statistical significance using t test analysis, *, p<0.05; **, p<0.01; ***, p<0.001.


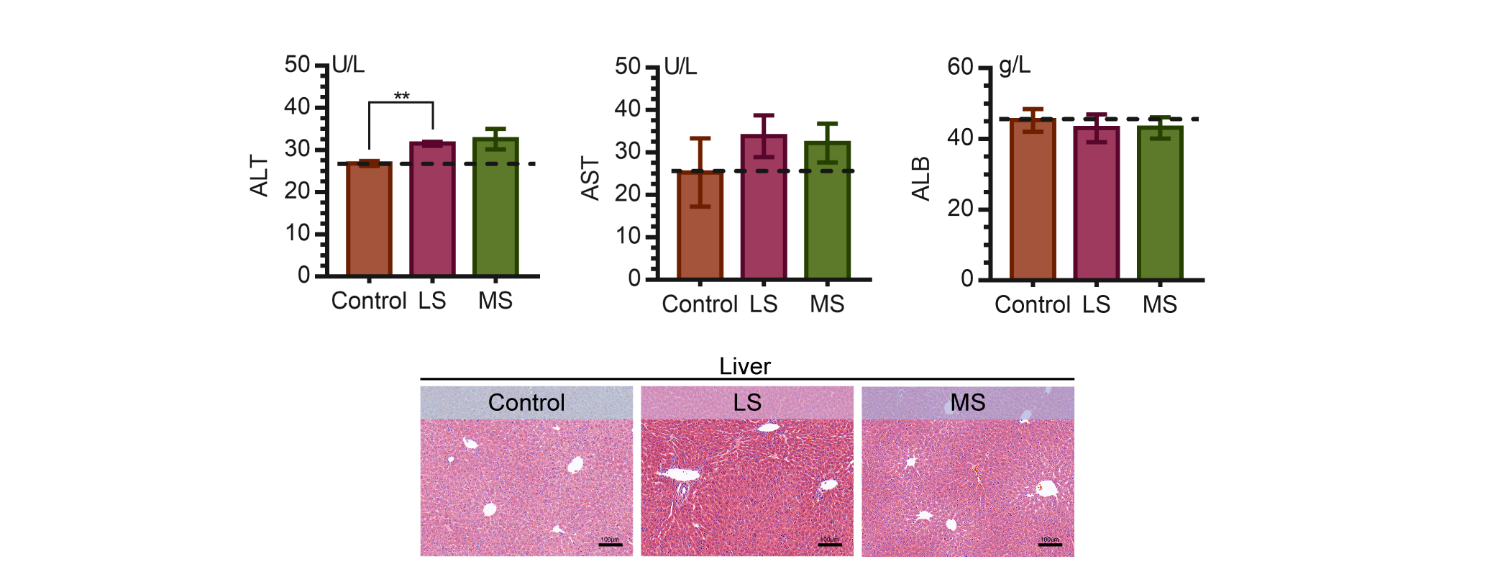


**Supplementary Figure 2.** Hepatotoxicity measured by ALT (alanine aminotransferase), AST (aspartate aminotransferase), ALB (albumin), and pathological section of liver (scale bar: 100 μm). The data were presented as mean±s.d. Difference between groups was examined for statistical significance using t test analysis, *, p<0.05; **, p<0.01; ***, p<0.001.


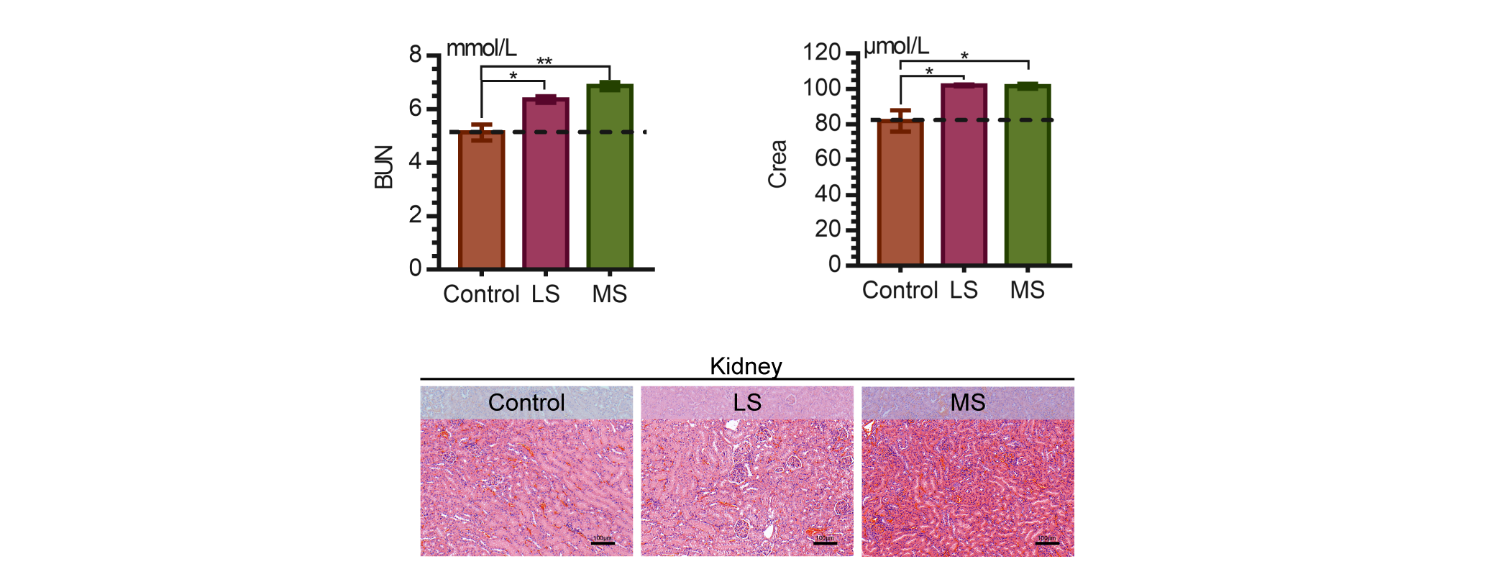


**Supplementary Figure 3.** Nephrotoxicity measured by BUN (blood urea nitrogen), Crea (creatinine) and pathological section of kidney (scale bar: 100 μm). The data were presented as mean±s.d. Difference between groups was examined for statistical significance using t test analysis, *, p<0.05; **, p<0.01; ***, p<0.001.


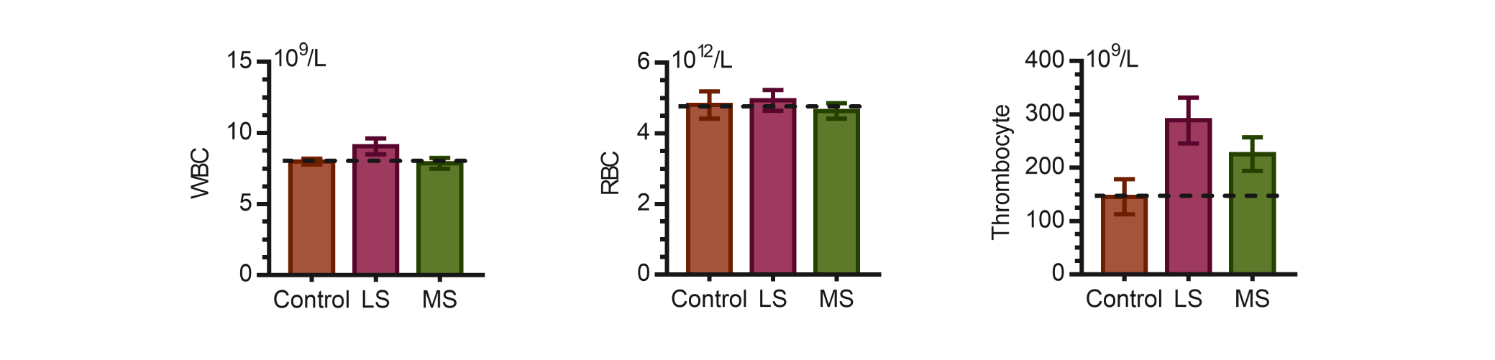


**Supplementary Figure 4.** Hematotoxicity measured by WBC (white blood cell) count, RBC (red blood cell) count and Thrombocyte count.


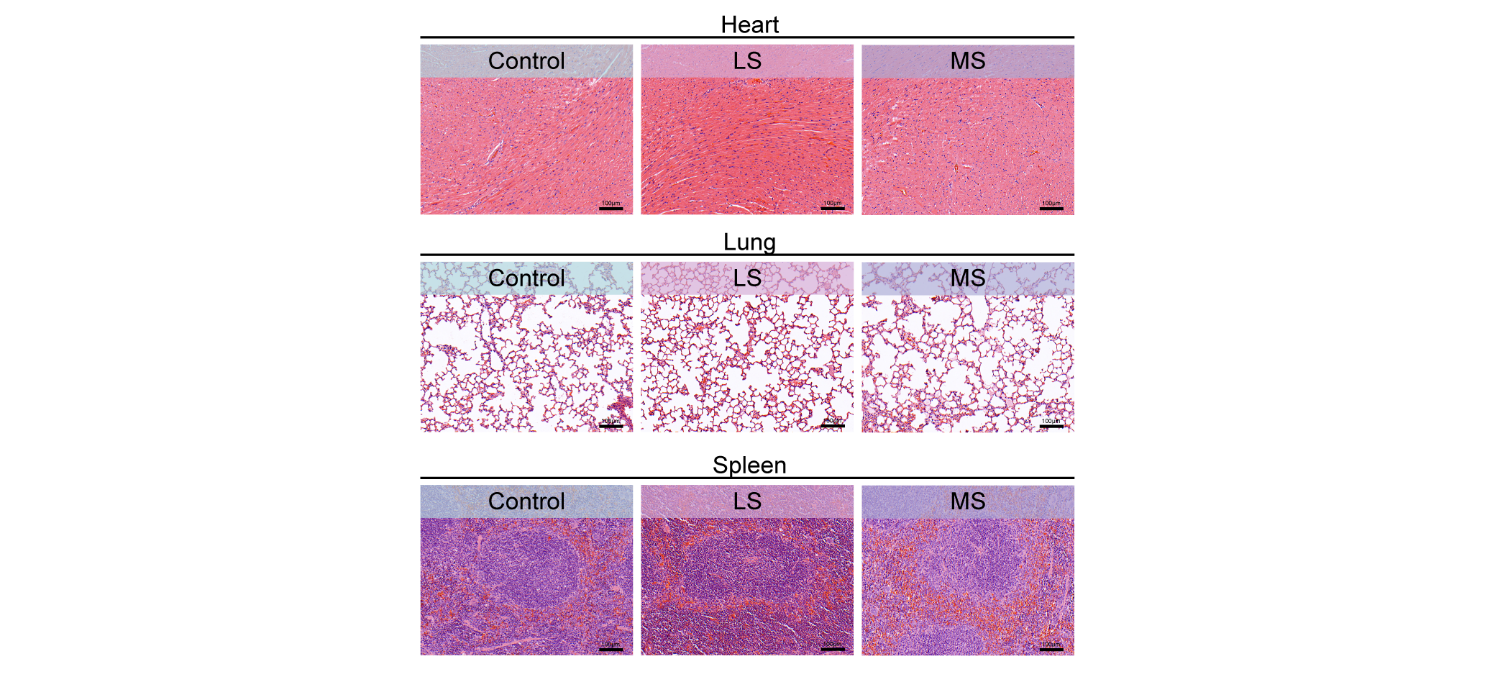


**Supplementary Figure 5.** Images of H&E-stained heart, lung, and spleen from mice after different specified treatments (scale bar: 100 μm).


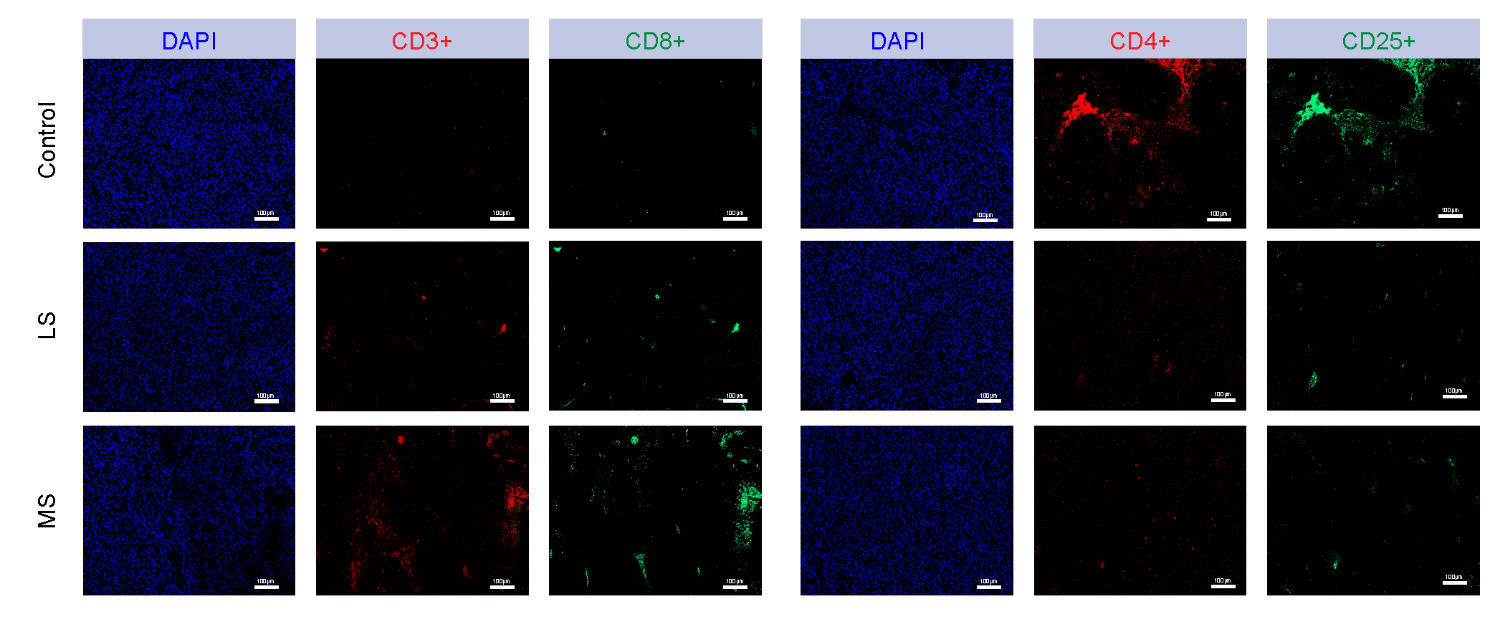


**Supplementary Figure 6.** Immunofluorescence images of CD3+, CD8+, CD4+ and CD25+ cells in tumor sections from mice after different specified treatments (scale bar: 100 μm).


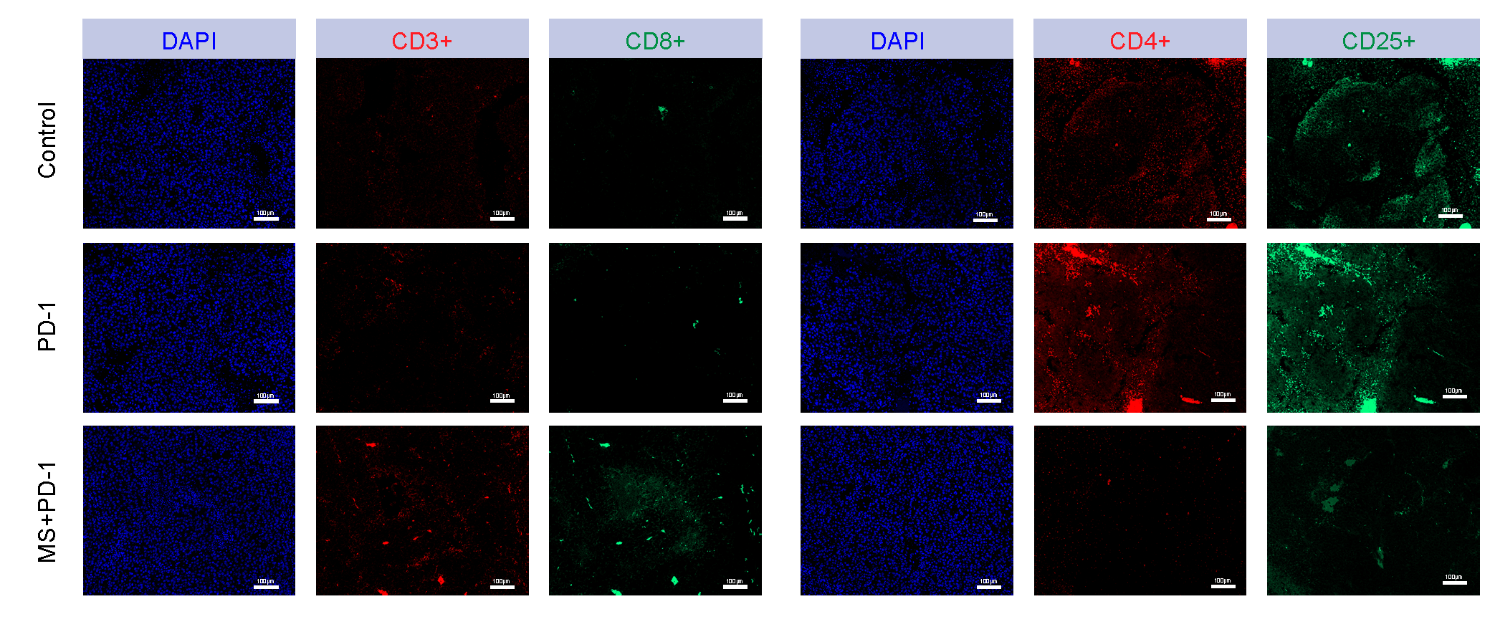


**Supplementary Figure 7.** Immunofluorescence images of CD3+, CD8+, CD4+ and CD25+ cells in tumor sections from mice after different specified treatments (scale bar: 100 μm).


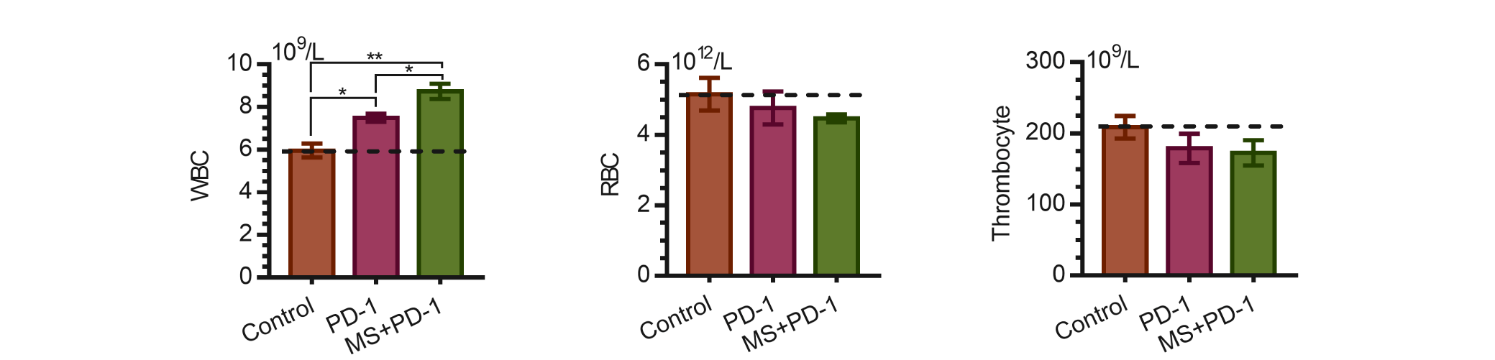


**Supplementary Figure 8.** Hematotoxicity measured by WBC (white blood cell) count, RBC (red blood cell) count and Thrombocyte count. The data were presented as mean±s.d. Difference between groups was examined for statistical significance using t test analysis, *, p<0.05; **, p<0.01; ***, p<0.001.


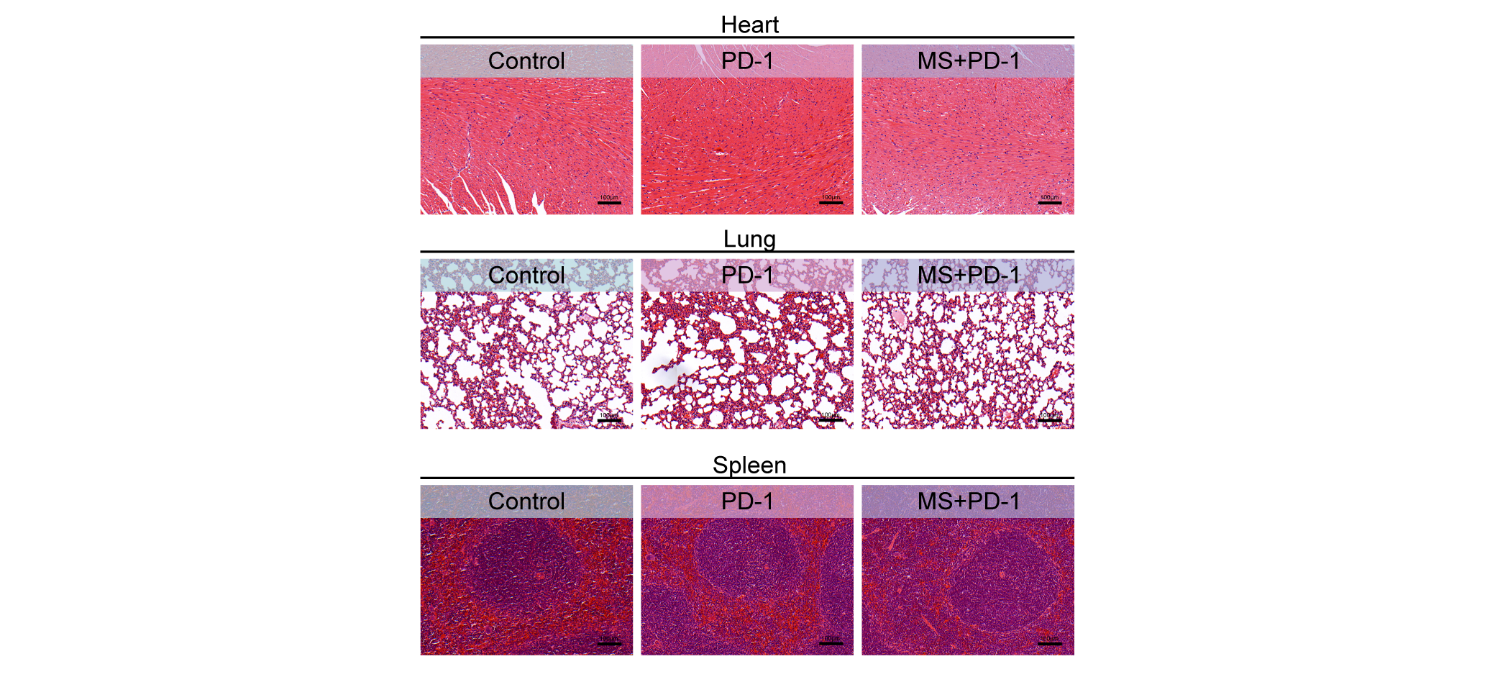


**Supplementary Figure 9.** Images of H&E-stained heart, lung, and spleen from mice after different specified treatments (scale bar: 100 μm).


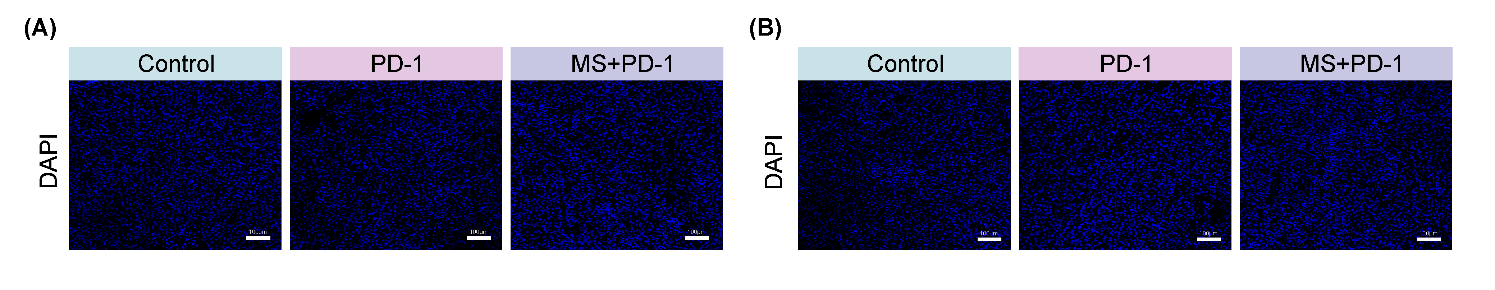


**Supplementary Figure 10.** (A) DAPI-separated images of Ki67 immunofluorescence in tumor sections (scale bar: 100 μm). (B) DAPI-separated images of TUNEL immunofluorescence in tumor sections (scale bar: 100 μm).

**Reference**

1. Jones JH. Resource Book for the Design of Animal Exercise Protocols. American Journal of Veterinary Research. 2007;68(6):583.

2. Bhandari V, Hoey C, Liu LY, Lalonde E, Ray J, Livingstone J, et al. Molecular landmarks of tumor hypoxia across cancer types. Nat Genet. 2019;51(2):308-18.

3. Buffa FM, Harris AL, West CM, Miller CJ. Large meta-analysis of multiple cancers reveals a common, compact and highly prognostic hypoxia metagene. Br J Cancer. 2010;102(2):428-35.

4. Yoshihara K, Shahmoradgoli M, Martinez E, Vegesna R, Kim H, Torres-Garcia W, et al. Inferring tumour purity and stromal and immune cell admixture from expression data. Nat Commun. 2013;4:2612.

5. Yang W, Liu W, Li X, Yan J, He W. Turning chiral peptides into a racemic supraparticle to induce the self-degradation of MDM2. J Adv Res. 2023;45:59-71.

6. Yan J, Zhang L, Li L, He W, Liu W. Developmentally engineered bio-assemblies releasing neurotrophic exosomes guide in situ neuroplasticity following spinal cord injury. Mater Today Bio. 2022;16:100406.

7. Wang J, Zheng X, Fu X, Jiang A, Yao Y, He W. A de novo dual-targeting supramolecular self-assembly peptide against pulmonary metastasis of melanoma. Theranostics. 2023;13(11):3844-55.
